# Academic retirement: A systematic review of the literature on retirement planning among university faculty

**DOI:** 10.1093/geroni/igaf122.4056

**Published:** 2025-12-31

**Authors:** Michelle Silver

**Affiliations:** University of Toronto, Toronto, Ontario, Canada

## Abstract

The overarching goal of this study is to inform institutional policy, support systems, and future research focused on enhancing retirement transitions within academia. In this scoping review we: (1) explore key factors influencing the retirement decisions of higher education faculty, including common pathways and barriers faced by faculty; (2) synthesize prominent study designs and findings in this area; and (3) evaluate the current state of the literature. A scoping review was conducted to address the outlined objectives. Relevant studies were identified through comprehensive searches across multiple academic databases using terms related to retirement decisions among university faculty. Four independent reviewers screened the literature and extracted key data using a standardized process to ensure rigor and consistency in study selection and synthesis. A total of 74 studies met the inclusion criteria. Most utilized cross-sectional survey designs, with qualitative interviews also commonly employed. Frequently examined variables included chronological age, partner or spousal retirement timing, health status, institutional policies, and professional identity. Geographical focus and academic subfields varied across the literature, revealing gaps in cross-contextual understanding. A concentration on individual and organizational factors was noted, with limited longitudinal research capturing transitions over time. This review builds on prior work by highlighting the varied nature of retirement among academic professionals, who often work in environments characterized by high autonomy. Understanding these retirement processes is critical for designing policies and practices that support career satisfaction and institutional knowledge retention. The findings have implications for retirement education and the development of inclusive retirement policies.

